# The Potential Role of Regional Anesthesia in Perioperative Anti-Inflammatory Treatments

**DOI:** 10.5812/aapm.5067

**Published:** 2012-07-10

**Authors:** Seyyed Hamid-Reza Faiz, Masood Mohseni

**Affiliations:** 1 Department of Anesthesiology, Rasoul Akram Medical Center, Iran University of Medical Sciences (IUMS), Tehran, Iran

**Keywords:** Regional Anesthesia, Inflammation, Vagus Nerve

We as regional anesthesiologists often use peripheral nerve blocks as a modality for intraoperative analgesia, but it is not well understood whether regional anesthesia can play a role beyond simple interruption of peripheral nerve conduction. Several published manuscripts in recent years on the potential non-analgesic applications of regional anesthesia encourage us to expand its role from intraoperative to the perioperative period. However, its clinical advantages are not fully disclosed and even its overall beneficial effect on patient outcome is still controversial (1).

The role of inflammation in the development of neuropathic pain has been explained earlier (2), which implies the therapeutic effects of sympathetic block in the treatment of chronic neuropathic pain disorders such as complex regional pain syndrome. The novel finding is the correlation between inflammation and the development of acute pain which has been proposed in a recent animal study (3). Regional anesthesia can play its anti-inflammatory role by anti-sympathetic effects (4) as well as anti-inflammatory properties of administered local anesthetics (5). However, the contribution of anti-inflammatory effects to overall clinical effects of peripheral nerve block is not well understood. This needs to be clarified in more meticulous trials addressing drugs and possibly techniques with more differentiated mechanisms of action. If elucidated, it may turn our views from simple interruption of impulse conduction in peripheral nerves to the possible anti-inflammatory mechanisms.

When reviewing the contribution of regional anesthesia to the postoperative pain and inflammation control, we should notice that ‘nerve block’ is just one side of the coin, while the other side, ‘nerve stimulation’ is even more interesting. Studies in recent years explain the role of vagus nerve stimulation (VNS) in the suppression of systemic inflammatory response known as ‘cholinergic anti-inflammatory pathway’ (6). It has been shown in animal studies that VNS reduces systemic levels of pro-inflammatory cytokines and Tumor Necrosis Factor (TNF) (7–9). This novel finding may have implications for postoperative period regarding the fact that uncontrolled inflammation plays an important role in the hyperglycemia, sepsis, shock and their consequent morbidity and mortality of critically ill patients.

VNS is currently used for its therapeutic effects in refractory epilepsy (10) and depression (11), and may be used for the treatment of chronic heart failure (12), portal hypertension (13) and eating disorders (14) in the future. Today application of VNS requires implantation of a stimulating electrode surgically (15). This procedure is invasive and has its specific complications (16, 17), which reduces its overall clinical benefit in the perioperative setting. However, favorable anti-inflammatory effects of VNS as well as its new potential perioperative applications such as therapeutic profile in the traumatic brain injury (18, 19) necessitates the development of a more feasible and less invasive approach to the nerve. Regarding the evolution in ultrasound imaging technology, it is expectable to better visualize the branches of vagus nerve and make them available for regional anesthesiologist with minimally invasive approaches. If so, this will expand the field of regional anesthesia from operating room to intensive care units in the near future.
